# Supplementary figures and images for: A population estimation study reveals a staggeringly high number of cattle on the streets of urban Raipur in India
Source: PLoS One. 2021 Jan 20;16(1):e0234594. doi: 10.1371/journal.pone.0234594 (PMC7817013; doi:10.1371/journal.pone.0234594)

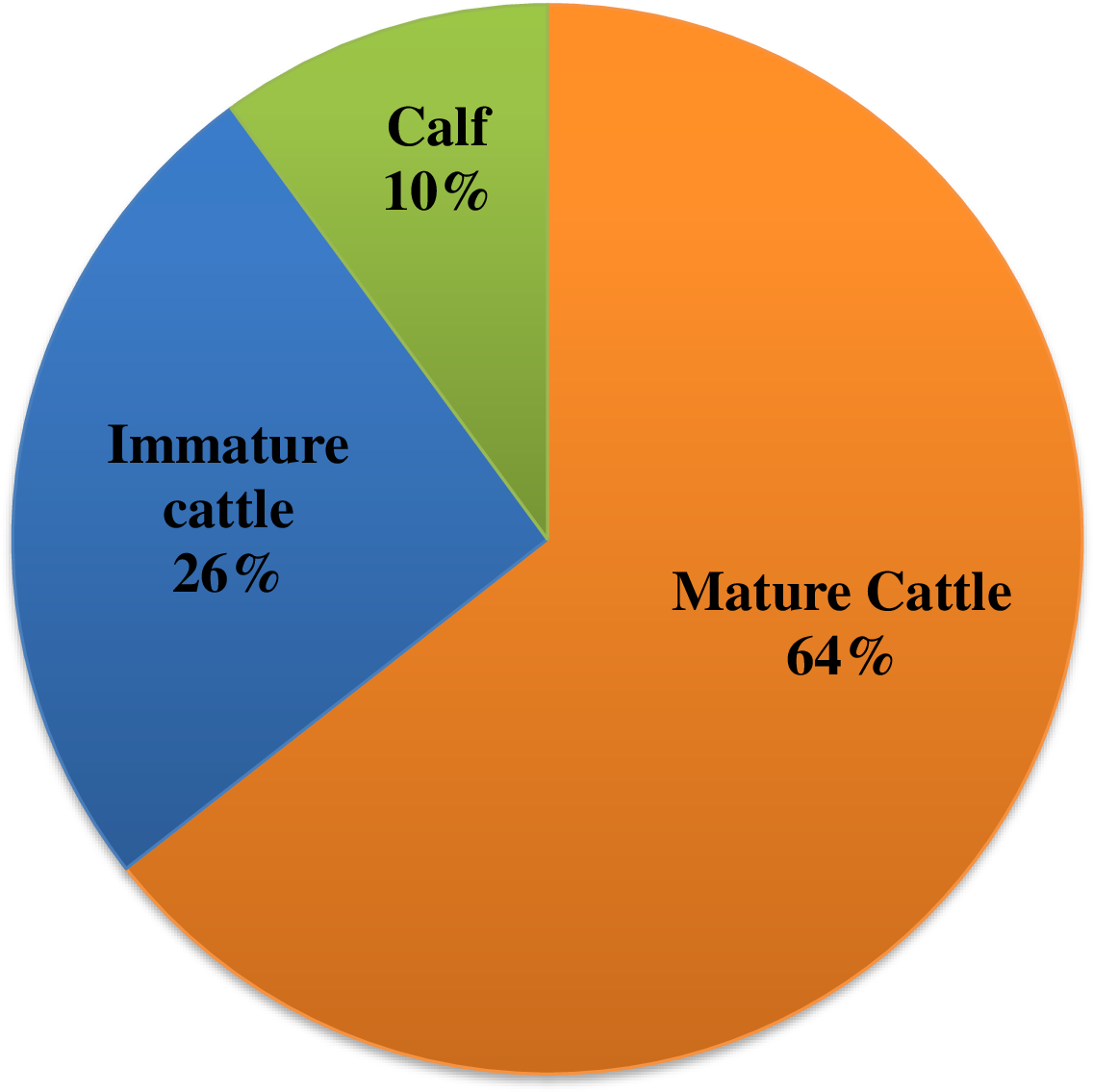

Supplement: S1 Fig — (TIF) [file pone.0234594.s001.tif]
