# Supplementary material for: A population estimation study reveals a staggeringly high number of cattle on the streets of urban Raipur in India
Source: PLoS One. 2021 Jan 20;16(1):e0234594. doi: 10.1371/journal.pone.0234594 (PMC7817013; doi:10.1371/journal.pone.0234594)
Supplement: S1 Table — (PDF) [file pone.0234594.s002.pdf]

S1 Table. Estimated population of street cattle per km distance after applying the data based on line transect sampling into the Lincoln index formula at 20 different sampling grids in Raipur city.

S. No.	Grid Number	Capturing Day (CD)	Recapturing Day (RCD)	Day 1 (cattle per km) n1	Day 3 (cattle per km) n2	Common in day 1 and day 3 (cattle per km) n3	Estimated cattle population (per km distance) N=n1×n2/n3
1.	83	14/02/2019	16/02/2019	14.71	11.55	3.72	45.61
2.	98	18/02/2019	20/02/2019	4.49	7.54	1.74	19.47
3.	101	22/02/2019	24/02/2019	21.41	15.26	8.88	36.79
4.	36	25/02/2019	27/02/2019	1.93	1.41	1.05	2.58
5.	27	25/02/2019	27/02/2019	11.89	7.77	1.58	58.24
6.	110	26/02/2019	28/02/2019	3.53	3.00	1.06	9.99
7.	94	09/03/2019	11/03/2019	5.86	9.88	1.23	46.91
8.	143	12/03/2019	14/03/2019	12.26	11.43	3.44	40.70
9.	115	13/03/2019	15/03/2019	14.24	14.24	6.07	33.40
10.	103	16/03/2019	18/03/2019	6.03	5.79	2.43	14.35
11.	89*	19/03/2019	21/03/2019	0.69*	0.12*	0.00*	undefined*
12.	32	20/03/2019	22/03/2019	6.57	4.83	1.61	19.71
13.	107	23/03/2019	25/03/2019	4.73	4.85	2.49	9.22
14.	76	26/03/2019	28/03/2019	4.95	4.56	1.07	21.16
15.	11	27/03/2019	29/03/2019	2.39	2.39	0.53	10.74
16.	1	30/03/2019	01/04/2019	1.54	1.69	0.28	9.27
17.	59	03/04/2019	05/04/2019	5.02	4.87	1.07	22.96
18.	135	04/04/2019	06/04/2019	7.32	8.65	3.22	19.68
19.	34	08/04/2019	10/04/2019	4.62	5.54	1.39	18.48
20.	70	09/04/2019	11/04/2019	10.74	10.27	4.62	23.90
Total estimated population in 19 km distance							**463.16**
Average density							**24.38**
Unbiased estimated population in 19 km distance				144.23	135.52	47.48	**411.67**[a]
Unbiased average density							**21.67**

[n1]Number of cattle per km distance photographed on the first day; [n2]Number of cattle per km distance photographed on the third day; [n3]Number of cattle per km distance sighted on the first day and also present on the third day; [N]Estimated population of cattle per km distance in 19 grids; [*]grid number 89 was not included in the calculation because the number of street cattle was undefined as per the Lincoln index formula when common street cattle in two sampling days is found to be zero, [a]unbiased estimation
