# Supplementary material for: A population estimation study reveals a staggeringly high number of cattle on the streets of urban Raipur in India
Source: PLoS One. 2021 Jan 20;16(1):e0234594. doi: 10.1371/journal.pone.0234594 (PMC7817013; doi:10.1371/journal.pone.0234594)
Supplement: S2 Table — (PDF) [file pone.0234594.s003.pdf]

S2 Table. Sex ratio of street cattle in different studied grid: sex ratio is the ratio of males to females in a population.

Grid ID	Grid Name	Average number of cattle		Sex ratio	Remark
		Male	Female		
83	Sarona	18	52.5	0.343	
98	Indraprasth Colony	13.5	28	0.482	
101	Daganiya colony	42	61.5	0.683	
36	WRS colony	1	8.5	0.118	Lowest
27	Bhanpuri	17	45	0.378	
110	Labhandi colony	6	12.5	0.480	
94	Jivan Vihar	6	19.5	0.308	
143	Bhatagaon	29.5	56.5	0.522	
115	Purani Basti	29.5	92.5	0.319	
103	Amin Para	17.5	33.5	0.522	
89	Jai Stambh Area	1.5	2	0.750	Highest
32	Sondongari	14	28.5	0.491	
107	Telibandha	10	28.5	0.351	
76	Shankar Nagar	20	29	0.690	
11	Kailash Nagar	7.5	10.5	0.714	
1	Transport Nagar	2.5	9	0.278	
59	Shivanand Nagar	12.5	20	0.625	
135	Mahaveer Nagar	22	50	0.440	
34	Govardhan Nagar	8	14	0.571	
70	Kota Colony	45	66.5	0.677	
**Total**		**323**	**668**	**0.484**	
