# Supplementary material for: A population estimation study reveals a staggeringly high number of cattle on the streets of urban Raipur in India
Source: PLoS One. 2021 Jan 20;16(1):e0234594. doi: 10.1371/journal.pone.0234594 (PMC7817013; doi:10.1371/journal.pone.0234594)
Supplement: Attachment — Submitted filename: Response to Reviewers.docx [file pone.0234594.s005.docx]

| **Action Taken Report**  **Manuscript Number: PONE-D-20-16241R1** | |
| --- | --- |
| **Response to Reviewer(s)' Comments** | |
| We would like to express our gratitude towards the esteemed reviewers for providing useful suggestions. We tried our best to revise the manuscript in compliance with the valuable tips offered to us. | |
| **Reviewer #1** | |
| **Reviewer comment 1** | Introduction L.91 - 100 Text added needs to be check for grammar and to be rephrased to be more sound. Any reference is missing. |
| **Author response 1** | We have rephrased the sentences to be more sound and have added the reference. |
| **Reviewer comment 2** | M and M Changes have been made accordingly L.246 ...formula in denominator maybe replace Total number by Total area (space) when measured in square km. |
| **Author response 2** | Corrections made |
| **Reviewer comment 3** | Conclusions Conclusion part was extended but from my point of view it is more extended summary than real Conclusions. I have to repeat that this is the weakest part of the manuscript. For innovation it is not enough to that mention ...this is the first study made.... Authors adopted some hints but ideas were not extended in Conclusions in meaning of broader usage nor made reader trust that the one of the methodologies used could be considered as "gold" standard or not. |
| **Author response 3** | We have further modified the section 'conclusions' in the revised manuscript. |
| **Reviewer #2** | |
| **Reviewer comment 4** | This is an improved manuscript, which reads well. I have a few very minor comments below, with the main ones about the conclusion. |
| **Author response 4** | We express our special thanks to the honorable reviewer to appreciate our revised manuscript. |
| **Reviewer comment 5** | Line 55- maybe saying the largest or highest zoomass may be better? |
| **Author response 5** | We have modified the statement as "In the animal world, cattle represent the largest zoomass." |
| **Reviewer comment 6** | Line 83- In contrast, a majority of the cows seen on…. (add in word) |
| **Author response 6** | Corrections made |
| **Reviewer comment 7** | Line 95- is Sri Lanka spelt as you have it? Sorry American English so just wanted to check |
| **Author response 7** | Corrections made |
| **Reviewer comment 8** | Line 416-417- a bit repetitive, consider deleting the last sentence |
| **Author response 8** | We have deleted the last repetitive sentence. |
| **Reviewer comment 9** | Line 430- the conclusion is still a bit of a summary- how does your study help to overcome human cattle interaction issues?  Why is the study important, could it help prevent interactions? Control disease? Be useful for population control etc? Things like that maybe worth mentioning. |
| **Author response 9** | We have further modified the section 'conclusions' in the revised manuscript. |

We have revised the manuscript, taking into consideration all inputs and suggestions of the esteemed reviewers. We do hope that the revised manuscript will be up to the expectations of the honorable reviewers and editors.
